# Development of a laser-based pre-damage for metal-polymer caps for sealing medical ampoules

**DOI:** 10.1038/s41598-025-32001-1

**Published:** 2025-12-08

**Authors:** Christoph Wortmann, Moritz Mascher, Stefan Behrens, Maximilian Brosda Flockenhaus, Alexander Olowinsky, Christian Hopmann

**Affiliations:** 1 Fraunhofer Institute for Laser Technology ILT, Steinbachstr. 15, 52074 Aachen, Germany; 2 Institute for Plastics Processing (IKV) in Industry and Craft, RWTH Aachen University, Aachen, Germany

**Keywords:** Joining polymers and metals, Laser-based structuring, Injection molding, Intended breaking forces, Hybrid-joined medical devices, Medical injection vials, Fibre lasers, Mechanical engineering

## Abstract

The closure of injection vials is realized by multi-material crimp caps consisting of aluminum and polymer. To improve the handling, previous investigations have determined which force for opening is perceived as comfortable and can be achieved with one hand. The potential of both material properties is utilized to develop a hybrid joining process which facilitates the opening of the vials. Undercut microstructures are introduced into the metal by laser radiation, which are filled by injection molding and ensure a microform-fit connection. This method leads to the elimination of adhesives and bonding agents, which require special regulations in medical technology. The aim of this research is to develop laser-based structuring for the aluminum sheet that ensures a reliable metal-polymer joint and breaks under a defined force. The challenge is to identify laser parameters that do not create a cutout or damage the thin aluminum sheet. A mechanical analysis is done by using a tensile testing machine in four different push-through arrangements to validate the opening forces. As a result, the targeted forces for single-handed opening of the injection vial are achieved. The metal-polymer joint remains connected while the flared cap is split open at an introduced pre-damage.

## Introduction

The combination of polymers with other materials, especially metals, offers many advantages such as weight reduction, cost efficiency and function integration. To achieve the advantages, a suitable joining process for joining the polymer component to the metal must be identified depending on the application and load case.

Joining polymers and metals is already used in a variety of medical device products, particularly in the packaging sector. The use of adhesives and bonding agents is often dispensed with, and form-fit joining methods are increasingly used. Due to the long innovation cycles in medical technology from ideas to market, medical products are often manufactured using outdated and disadvantageous manufacturing processes. As part of a project funded by the German Federal Ministry of Education and Research (BMBF) (grant no. 03XP0291), a low-cost, automated production line for the manufacture of medical caps on glass ampoules was developed.

**Fig. 1:**
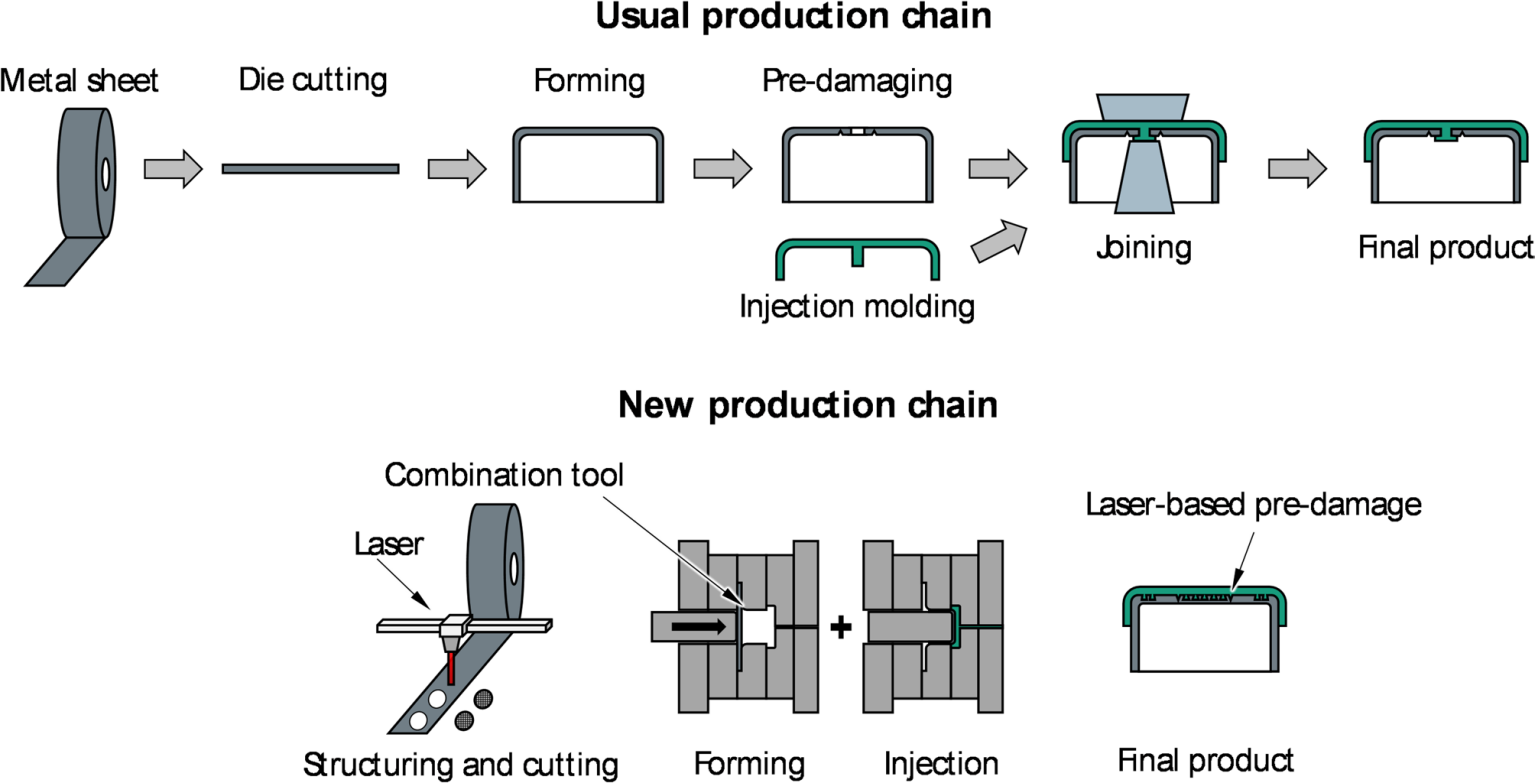
Comparison of the two production chains for manufacturing crimp caps.

Previously, the aluminum coil was processed in a stamping, forming, and pre-damaging step^1^. The two components are then joined with an injection-molded polymer cap^2^. The developed process chain in this project combines several processing steps in one laser system. An aluminum coil is passed through the laser’s processing area for micro-structuring, pre-damaging, and cutting of the circular blank from the aluminum. Optionally, the product can be labelled using a laser. In a second step, the blank is formed into a lid in a developed combined deep-drawing/back injection mold and back-injected with polymer. The polymer penetrates the microstructure created by the laser, which leads to a form-fit bond between the polymer and the metal. Figure 1 provides an overview of the two production chains.

Conventional joining techniques, such as adhesive bonding, necessitate sophisticated process control and prolonged curing times, and they are often difficult to disassemble^3–6^. In medical technology in particular, adhesives are associated with additional certification costs. Screw connections necessitate auxiliary elements and induce pronounced stress concentrations, thereby creating weak points within the material^5,7,8^. A reliable aluminum-polymer joining method is a laser-based, two-step approach in which the metallic component is first microstructured, followed by thermal bonding of the polymer component^6,9,10^. Many publications on laser-based structuring of metals have been published to date. A distinction is made between laser structuring with continuous wave lasers (CW) and pulsed lasers. CW lasers offer the advantage of a high ablation rate with reproducible cavity geometries. Investigations with pulsed lasers show that a high structure density and magnificent local accuracy can be achieved^11^. Various surface structures applied by laser radiation were investigated and then joined with a polymer component^3,9,11–14^. Studies have examined the wettability of microstructures, yielding insights into subsequent polymer joining behavior^3,15^. Additionally, laser-based metal-polymer joining and its influence on mechanical strength have already been investigated^11,16,17^. Various joining techniques, including adhesive bonding and induction joining, were benchmarked against laser-based joining with respect to mechanical strength^9,18^. Studies have also been published on the bonding of polymer and metal via in-mold assembly in the injection molding process^19–24^. To increase the bond strength of the in-mold assembly, the surface of the metal component can be structured using laser radiation^25–28^. Likewise in this research, the polymer is injected onto the laser-structured aluminum in an injection molding machine. The focus of the work is the laser-based pre-damaging of the aluminum cap for standardized opening of the ampoule.

**Fig. 2:**
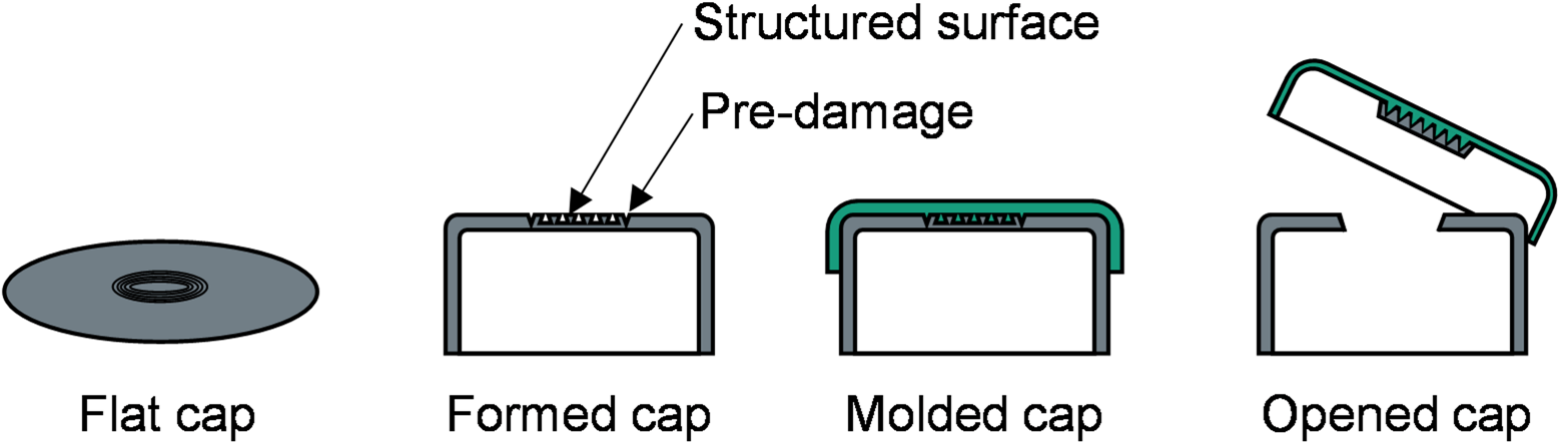
Shaping of the caps along the process chain.

Laser pre-damaging of the aluminum is ambitious to achieve the desired opening forces according to the standard DIN EN ISO 8362^29,30^. In addition, the bond strength between the metal and polymer is fundamental to the further handling of the vial. Detaching the polymer component from the metal cap at the site of the circular pre-damage requires complete tearing of the inner bonding surface, creating a hole in the cap that allows access to the contents of the vial. Figure 2 illustrates the detachment of the polymer cap along with the pre-damaged aluminum component.

In addition to the injection molding and material parameters of polymer and aluminum, the process parameters of laser structuring also influence the bond strength. In this paper, the process parameters for laser-based pre-damage are scientifically investigated and the effects of external influences on the subsequent opening forces are examined in more detail. This results in the research question: How can the aluminum be pre-damaged with laser radiation in such a way that the desired opening forces of the vial are not exceeded, while at the same time maintaining a positive bond between metal and polymer in the central area of the crimp cap? The aim of this work is to find suitable parameters for the laser pre-damage of the aluminum to ensure the desired handling of the medical vial and not to exceed the maximum opening force. External influences like a sterilization process on the opening forces will also be investigated to validate the process for the subsequent use.

## Materials and methods

In the first step the surface of the aluminum is structured, pre-damaged and then cutted. For this purpose, a 2 kW fiber laser from Coherent namely a Rofin FL 020 C laser (λ = 1070 nm) is used. The optical system comprises a Scanlab intelliSCAN 20se galvanometric scanner providing x-y beam deflection, coupled to a Sill Optics F-Theta lens (Q 340/20; S4LFT1330/328) that delivers a flat-field focus on the processing plane. The resulting focal spot diameter is 38 μm. The metal component is structured by focusing the laser beam onto the aluminum surface, where irradiation locally heats the metal, causing melting and vaporization. The ensuing vapor pressure expels the melt, which partially resolidifies at the neck of the developing cavity. Galvanometric scanning across the surface forms trench-like cavities. Multiple passes at the same location increases the structure depth, and undercuts form the laser-structured surface. The structuring process is also used for pre-damaging, but the laser applies more energy locally than in the structuring process. This creates a deep cavity in the aluminum that does not extend through the entire sheet and thus serves as a pre-determined breaking point. The final circular cut of the blank from the sheet is based on the same fundamental principle. The material utilized is EN-AW 8011 A aluminum, which has been approved for use in medical technology and possesses a thickness of 200 μm and a strength of H14. The chosen polymer is PP PCGR40, which is also in compliance with the regulations for its use in the medical and pharmaceutical industries.

**Fig. 3:**
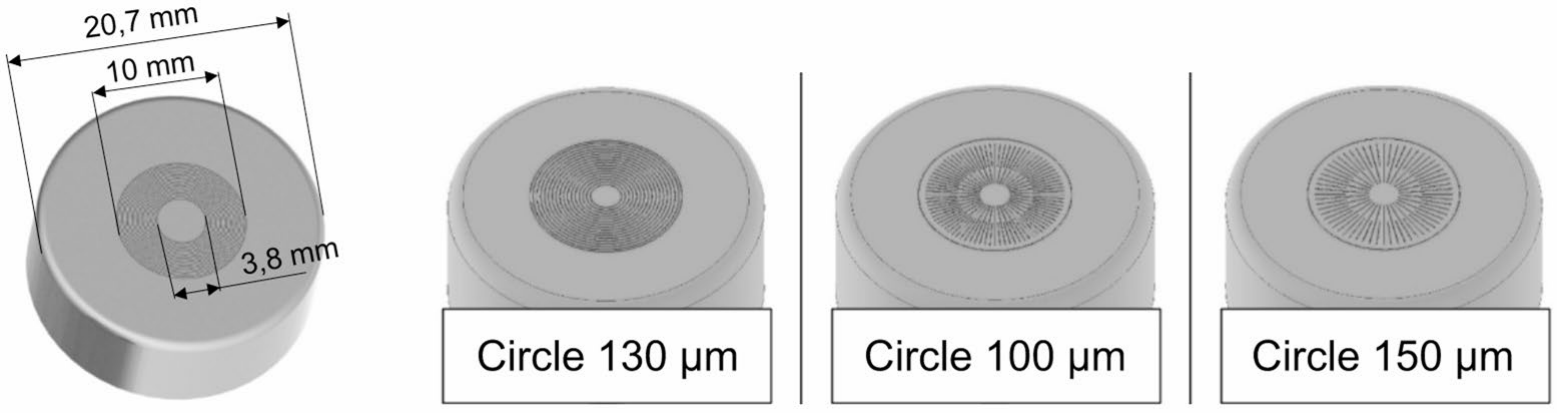
Different laser-structured geometries for maximum bond strength.

In the preliminary tests, experiments are conducted to determine the optimal geometry and size of the structured metal surface on the samples. The objective is to utilize as much of the pre-damaged area as possible to achieve the highest possible bond strength between metal and polymer. For this purpose, different geometries (for example, star-shaped or circular) were tested. Figure 3 illustrates the geometries examined for the laser-structured surface.

**Fig. 4:**
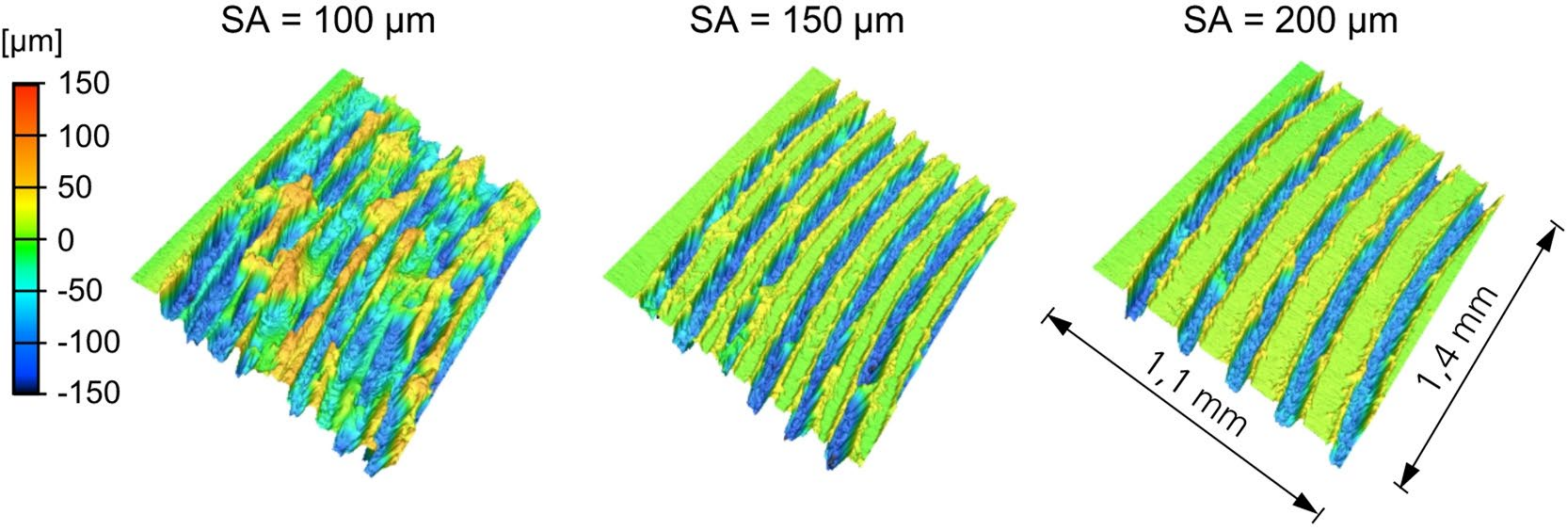
Different structure distances on the aluminum surface.

The results indicated that the surface should be structured in a circular manner to achieve the largest cumulative structure length. To strike a balance between the highest possible utilization of the 10 mm diameter surface and reproducibility, the structure distance between the circular structures was determined through iterative testing. The area in the center (diameter less than 3.8 mm) of the cap was not structured, since the small radius requires a lower scanner speed which leads to non-reproducible cavities. The laser parameters to create the structure are developed by analyzing cross-sections. As a result, the aluminum is structured with a laser power of *P* = 1.0 kW, a scanner speed of v = 15 m/s, and *N* = 2 passes. Figure 4 illustrates different structure distances on the surface using a laser scanning microscope.

Insufficient structure distance between leads to overlaps of melting deposits, thereby reducing reproducibility and bond strength. Conversely, excessive spacing underutilizes the available area within the 10 mm circular region, which likewise diminishes bond strength. Accordingly, a structural distance of SA = 130 μm was selected as a compromise.

**Fig. 5:**
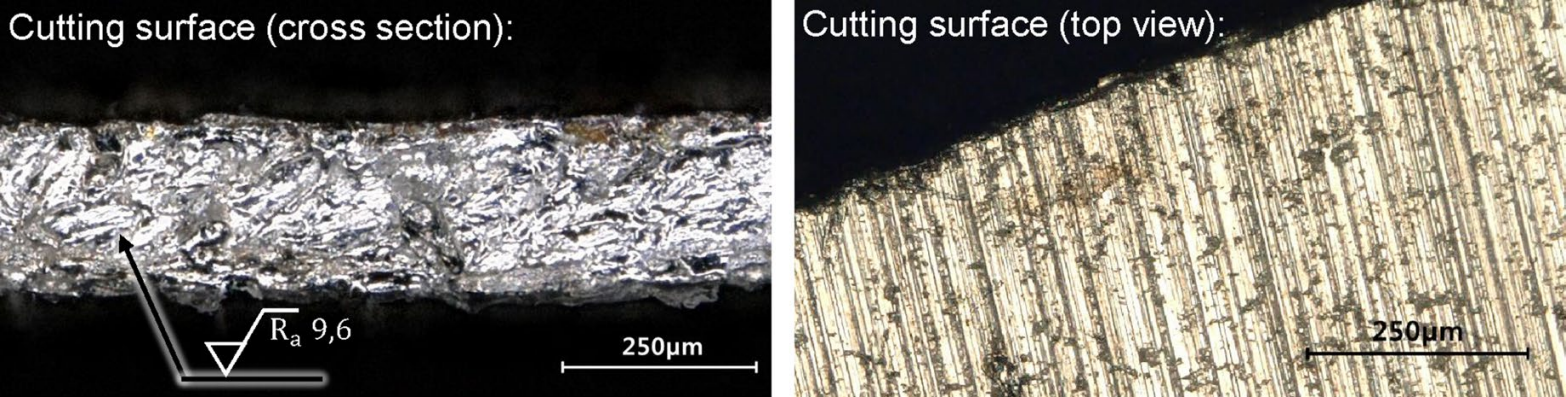
Detailed view of the cut surface of the cap (cross section left and top view right).

To cut the circular blank from the aluminum sheet, *N* = 14 passes with a laser power of *P* = 1.0 kW and a scanner speed of v = 15 m/s are used. The outer diameter of the cut blank is 31.7 mm. The pre-damage is applied with a diameter of 10 mm so that the subsequent opening of the cap has the same diameter^29^. Figure 5 illustrates a detailed view of the cut surface. Subsequent characterization revealed an average cut-surface roughness of Ra = 9.6 μm, which is adequate for the intended application. Although surface quality could be further improved by pulsed laser systems, such optimization was beyond the scope of this study.

**Fig. 6:**
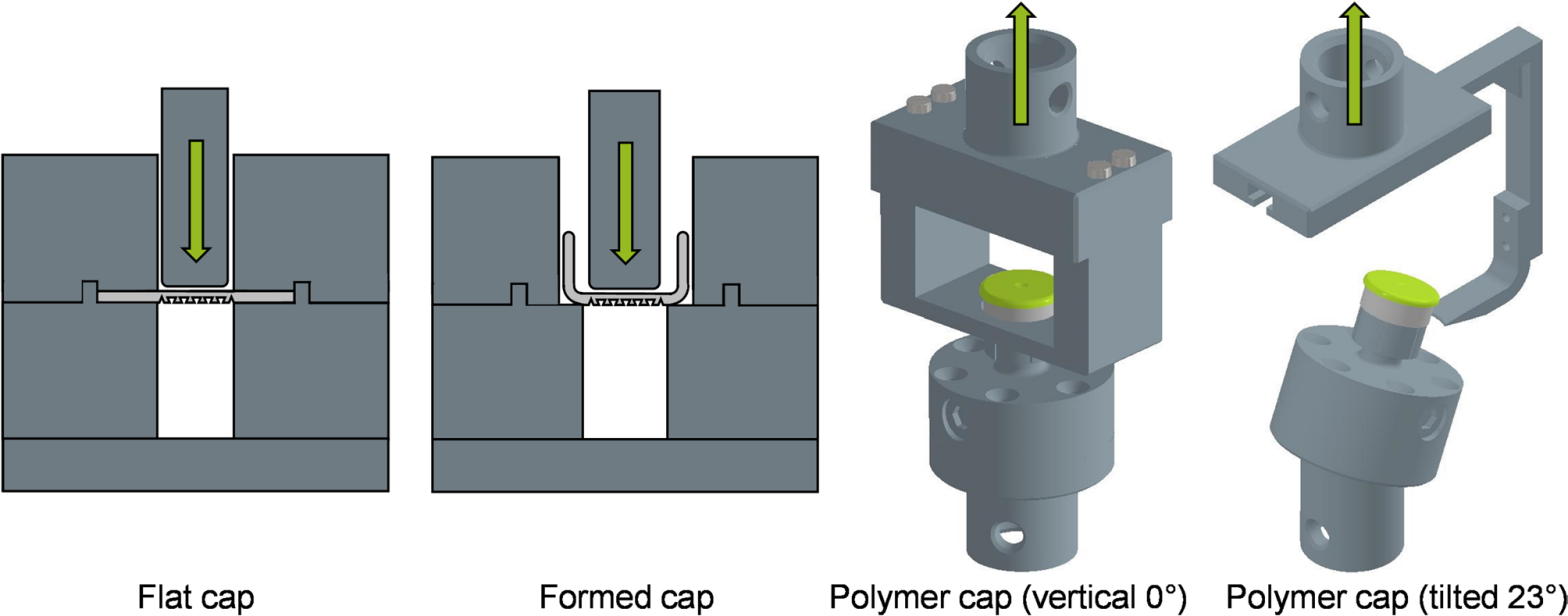
Four test arrangements during push-through and tensile tests of the different caps.

After laser structuring, pre-damaging, and cutting, the blanks are deep drawn in an injection molding machine. The polymer is then injected to create a form-fit bond in the structured area. The injection molding process is carried out at the Institute for Plastic Processing (IKV) at RWTH Aachen University. The used injection molding machine is KraussMaffei Technologies 110–380 CX. The combination mold was developed by Siegfried Hofmann GmbH. Injection molding parameters such as injection speed (20 cm^3^/s), holding pressure (100 bar), mold temperature (60 °C) and melt temperature (300 °C) are kept constant^31^. The microstructure filling and bond strength of the joint can be significantly influenced by the injection molding process. The selected process parameters were determined by means of a comprehensive process analysis. High bonding forces between the polymer and the aluminum sheet that exceed the force required to open the pre-damage, ensure a safe cap opening. For the mechanical analysis of the laser-based pre-damage, the aluminum components are tested in four different shapes in a tensile testing machine. Figure 6 shows an overview of the four test arrangements.

To investigate external influences on the opening forces of the molded and deep-drawn caps in angled test configuration, some of the caps are subjected to climate change tests and sterilization tests. Temperature changes can place a high load on plastic/metal hybrid components due to the different coefficients of thermal expansion. The influence of crimping on the bond strength was also investigated. Commercially available crimping pliers are used to crimp the caps onto a medical injection vial. A ClimeEvent C/180/40/3 climate chamber from Weiss Technik GmbH, Reiskirchen, Germany, is used for the climate change and sterilization tests. The climate change test is conducted in accordance with the PV 1200 protocol^32^. A total of 17 climate change cycles are performed. The sterilization test is conducted in accordance with the standards in DIN EN ISO 8872^33^.

## Results and discussion

**Fig. 7:**
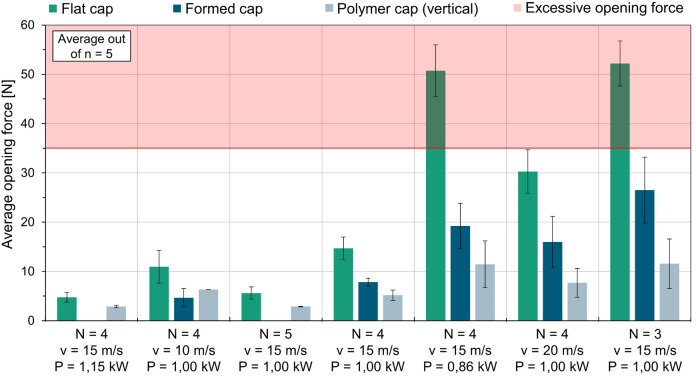
Results on opening forces due to pre-damage by laser (test series 1).

Approximately 300 parameter sets for laser-induced pre-damage were analyzed in this study. However, two test series are discussed in detail. The first series was used to narrow down the laser parameters, whereas the second presents the key findings. Within each series, laser power, scanning speed, and number of passes were systematically varied to quantify their influence on opening forces. In the first test series, seven different parameter sets were examined for possible pre-damage, which must not exceed a force of 35 N in accordance with the DIN EN ISO 8362 standard for crimp caps. At the same time, to prevent the composite from failing during transport, the opening force must not be too low. Starting from a central parameter, the laser power, scanner speed and number of passes were varied upwards and downwards so that greater and lesser pre-damage occurs. The result is shown in Fig. 7.

**Fig. 8:**
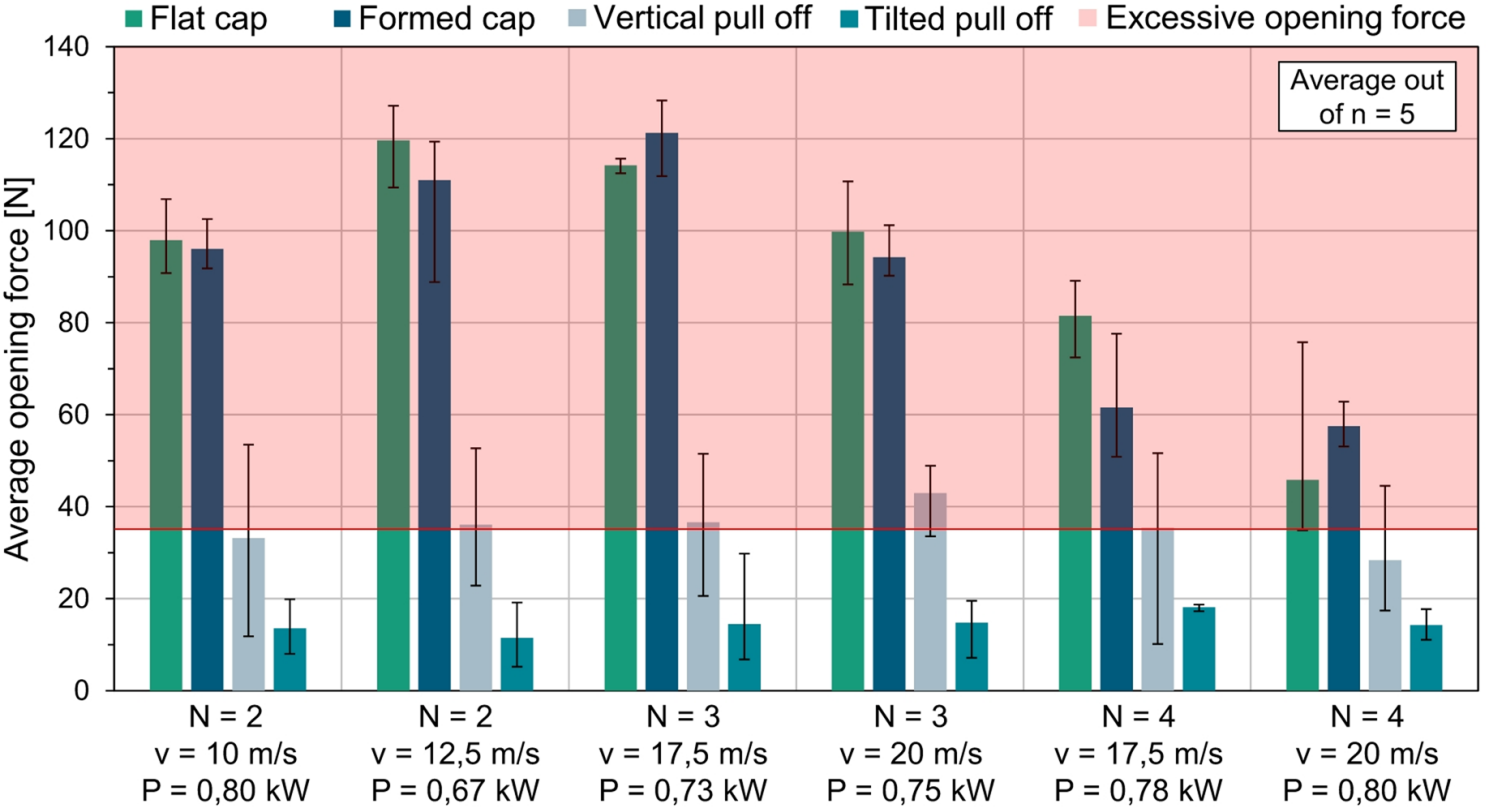
Results on opening forces due to pre-damage by laser (test series 2).

Figure 7 illustrates the average opening forces from five identical samples for the respective laser parameters. As part of the first test series, the tests were conducted on three cap shapes: flat caps, formed caps, and caps molded with polymer (in vertical pull off). The central parameter with a laser power of *P* = 1.00 kW, a scanner speed of v = 15 m/s at *N* = 4 passes is shown in the middle. With an average opening force of 15 N, the flat cap is below the opening force required by the standard. For the other cap shapes with the same laser parameters, the opening forces decrease with each subsequent process step. The average opening force for the deep-drawn cap is approximately 8 N. The average opening force in the vertical pull off test is 5 N for the deep-drawn cap molded with polymer. It can be assumed that the laser-based pre-damage in the flat blank is intensified in this test series by the deep-drawing process. The material parameters show that the aluminum expands only 2% before it breaks. Due to the internal stresses introduced during the forming process, mechanisms seem to occur on the ground of the cavity, which is used as the pre-damage. This can result in higher stresses or micro-cracks in the notch base. This contributes to an early failure of the aluminum, resulting in significantly lower average opening forces for the formed cap. During the injection of the polymer into the cavities, the formed cap is subjected to thermal and mechanical stress. The cap is heated by the preheated mold in which it is placed during the injection molding process. As aluminum expands as the plasticized polymer flows in with high pressure, and, based on the material data, hardly deforms before breaking, a crack is expected to occur in the cavity base. Furthermore, the demolding after injection molding leads to a mechanical load on the cap. This mechanism increases the pre-damage, so that the opening forces are reduced. These observations can also be seen for other laser parameters for the molded caps. By reducing the laser power to *P* = 0.86 kW while keeping the scanner speed and the number of passes constant, less material is vaporized from the cavity used as the pre-damage, so that the depth of the pre-damage and its notch effect decreases. This increases the average opening force for the flat caps to 51 N. A significant reduction in the opening forces due to the forming step and injection molding with polymer can also be observed with this parameter set. Increasing the scanner speed to v = 20 m/s reduces the introduced energy density into the aluminum per pass of the scanner around the pre-damage, while the laser power remains constant. The reduction in line energy results in a decrease in the depth of the cavity, which serves as the pre-damage. This is illustrated in Fig. 7, which shows an increased average opening force of F = 30 N for the flat caps. The opening forces also decrease during the deep-drawing and injection molding process, as shown by these parameters. Reducing the number of scanner passes to *N* = 3 also reduces the formation of the cavity in terms of shape and depth and, in the case of pre-damaging, leads to an increase in the opening forces to F = 52 N. The opening force drops to 26 N for the formed caps and 12 N for the molded caps. The parameters used in the first test series, which were intended to achieve greater pre-damage than the central parameter, showed very low opening forces. Since there were not enough samples available for evaluation with these parameters and they are also well below the opening forces targeted by the standard, this is not explained separately. Consequently, a second test series was devised based on the previous results. The objective was to enhance the opening forces, especially those for the molded caps tested in 23° alignment, to approach the forces set in the standard. The outcomes of the second test series are presented in Fig. 8.

As part of the second test series, investigations were carried out on four different cap shapes: a flat cap, a formed cap, and caps injection-molded with polymer (in 0° vertical and 23° tilted take-off tests). Based on the central parameter of the first test series, which was *N* = 4 passes, v = 15 m/s, and *P* = 1.00 kW, the energy input for the pre-damage was reduced to achieve a higher opening force. The average opening forces from five identical samples shown in . 8 represent only a portion of the investigation carried out.

The line energy of the two left-hand parameters with *N* = 2 passes and laser powers of *P* = 0.80 kW and *P* = 0.67 kW is significantly below the central parameter of the first test series. For a scanner speed of v = 10 m/s, this results in an average opening force of around 100 N for the flat cap and formed cap, and of 110 N to 120 N for a scanner speed of v = 12.5 m/s. Despite the lower speed of the two parameter sets, the number of laser passes and the lower laser power compared to the central parameter result in significantly less pre-damage. This is due to the significantly lower line energy of E = 160 J/m for the parameters with v = 10 m/s and E = 107 J/m for the parameters with v = 12.5 m/s scanner speed. The line energy of the central parameter is E = 267 J/m, which leads to a more pronounced pre-damage cavity. For the parameters with *N* = 2 passes in . 8, the previous observation that the deep-drawing process has an influence on the pre-damage does not apply here. The opening forces of the formed cap are within the same range as those of the flat cap. It can therefore be concluded that the deep-drawing process does not initiate cracks at the base of the pre-damage cavity. However, as previously observed, there is a considerable drop in the opening forces due to the injection molding process with polymer, which occurs due to the thermal and mechanical stress on the cavities caused by the inflow of the plasticized polymer. A lower average opening force is observed in the tilted pull off (23°) than in the vertical pull off test (0°). This is attributed to the distinct type of load on the pre-damage. In the tilted pull off, a peel load is present around the pre-damage, while a tensile load is observed in the vertical pull off. The distribution of stress in a peeling load is significantly higher than in a stress distribution that is evenly distributed over the surface. Consequently, failure occurs at lower forces in the 23° tilted test arrangement.

The pre-damage of the two parameters shown in the middle of . 8 with *N* = 3 passes is generated with a similar laser power. The difference between the two parameters is the scanner speed, which leads to a distinction in the applied line energy. For a scanner speed of v = 20 m/s, the total line energy is E = 113 J/m. For a scanner speed of v = 17.5 m/s, the line energy is E = 134 J/m. This difference is demonstrated by lower opening forces. It is also evident that the injection molding process results in a reduction in opening forces for the molded caps, due to the introduced tensions. The discrepancy between the two test conurations of the molded caps is again explained by the disparate stress distributions.

The two parameters shown in the right-hand section of . 8 are characterized by *N* = 4 passes. Despite high scanner speeds, the line energy is higher than in the parameter sets previously examined in the ure, which is shown by lower opening forces. Particularly noteworthy here is the highest opening force of F = 19 N in the tilted pull off test. Although no formal minimum opening force is specified, excessively low opening forces may cause pre-existing damage to reopen under rough handling. Accordingly, an opening force close to 35 N is recommended as defined in DIN EN ISO 8362 standard.

The results for the average opening force were measured closely after production of the respective cap shape. The situation is different if the caps are used to close medical injection vials. In this case, they are crimped onto the bottle head and subjected to a sterilization process. Depending on the type of storage, transportation and duration until final use, different climatic conditions may prevail. ure 9 uses a reference cap to illustrate the external influences on the opening force.

**Fig. 9:**
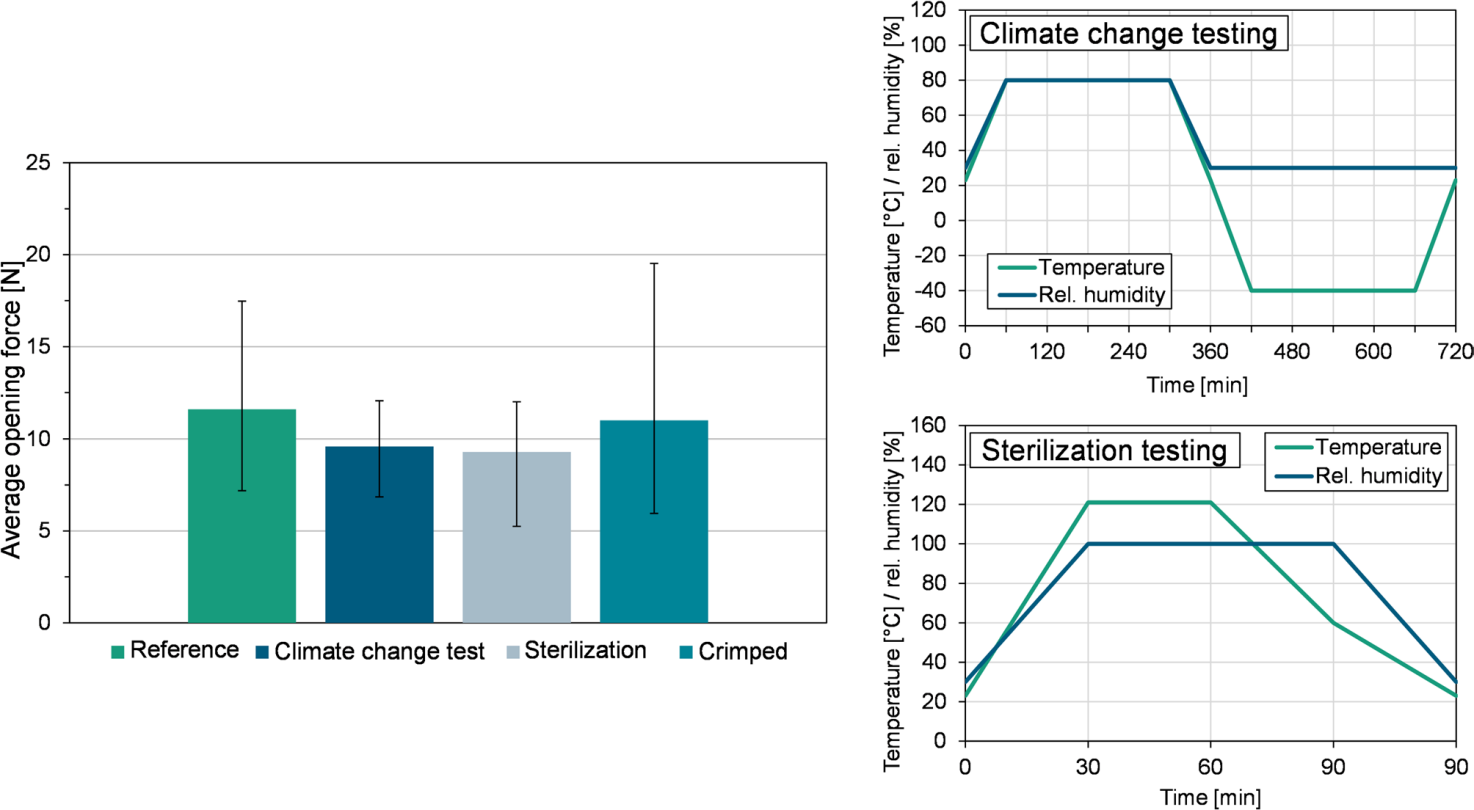
External influences on the opening forces of polymer molded caps (tilted test).

The examined caps in Fig. 9 were tested in the 23° tilted test arrangement. Despite the considerable standard deviation, it can be observed that the crimping process, climate change test, and sterilization have no significant impact on the pre-damage. One hypothesis was that the crimping process would exert mechanical stress on the pre-damaged material, thereby decreasing the opening forces. However, this was not borne out by the results of the second test series for the formed caps. Climate change tests and sterilization have a thermal effect on the caps. Due to the different thermal expansion of the aluminum and polymer during a temperature change, stresses occur in the contact surface between the two materials. These tensions show a slight decrease in the average opening force. One potential explanation for this phenomenon is that the thermal expansion of aluminum during heating causes a contraction in the cavity, particularly in the pre-damage region. This contraction exerts pressure on the polymer within the cavity, which, if not inhibited, can lead to micro-damage in the base of the pre-damage region. To gain a more comprehensive understanding of this hypothesis, further in-depth investigations are necessary.

## Summary and outlook

The investigation of the opening forces through the laser-based pre-damage of medical crimp caps demonstrated promising results. In the first test series, the laser power, scanner speed, and number of passes were varied based on a central parameter to approximate the desired opening force of 35 N maximum. As part of the second test series, a comprehensive investigation was conducted. Some of these results were presented in this paper. These results demonstrated a good approximation of the maximum opening forces specified in the standard. A laser power of *P* = 0.78 kW, a scanner speed of v = 17.5 m/s, and *N* = 4 passes resulted in an opening force of approximately 19 N in the tilted pull off test. An aspect of the overall results was the reduction in opening forces that resulted from injection molding of the polymer. This is caused by the thermal and mechanical stress of the pressed-in and plasticized polymer during the joining process, which increases the pre-damage. The discrepancy between the vertical and tilted cap pull off was attributed to the unequal stress distribution caused by the two types of stress in the contact zone. Furthermore, no influence of external influences such as climate change tests, sterilization, or crimping of the crimped cap on the opening forces can be determined. In further investigations, the mechanical stress on the cavity caused by the inflow of plasticized polymer during joining should be examined and possible damage mechanisms identified. The influence of the injection molding parameters on the opening forces should also be investigated in more detail to minimize the drop in forces. Finally, it is recommended that analyses be conducted to determine the media tightness of the caps to prevent the penetration of foreign substances into the injection vial.

## Data Availability

The datasets used and/or analysed during the current study available from the corresponding author on reasonable request.
